# Supplementary figures and images for: Formation of multinucleated osteoclasts depends on an oxidized species of cell surface-associated La protein
Source: eLife. 2024 Oct 2;13:RP98665. doi: 10.7554/eLife.98665 (PMC11446546; doi:10.7554/eLife.98665)

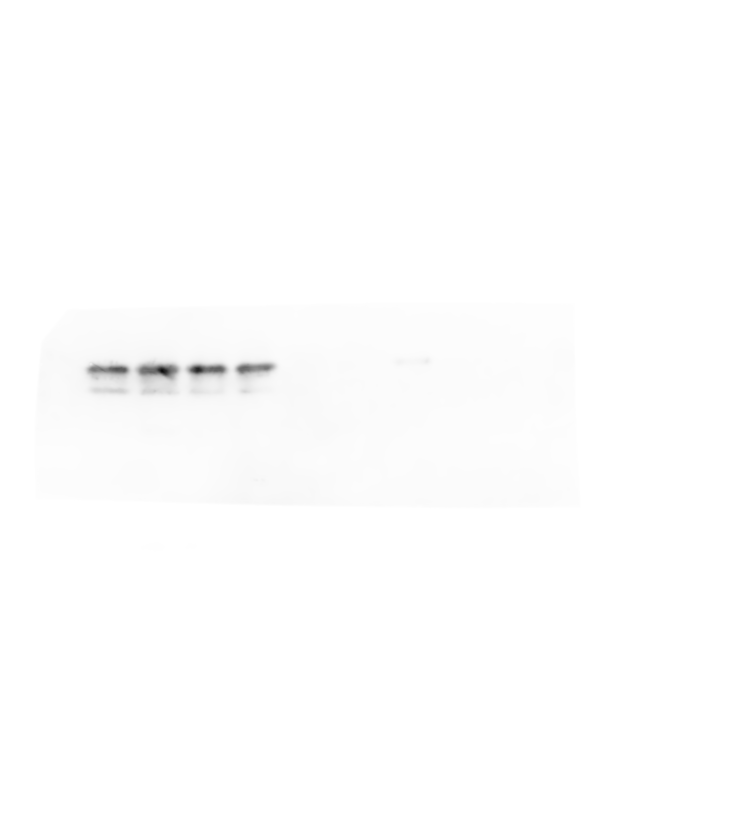

Supplement: Figure 4—source data 2. [file elife-98665-fig4-data2.zip › Figure 4-Source Data 2/Figure 4-Raw Gels-B.tif]

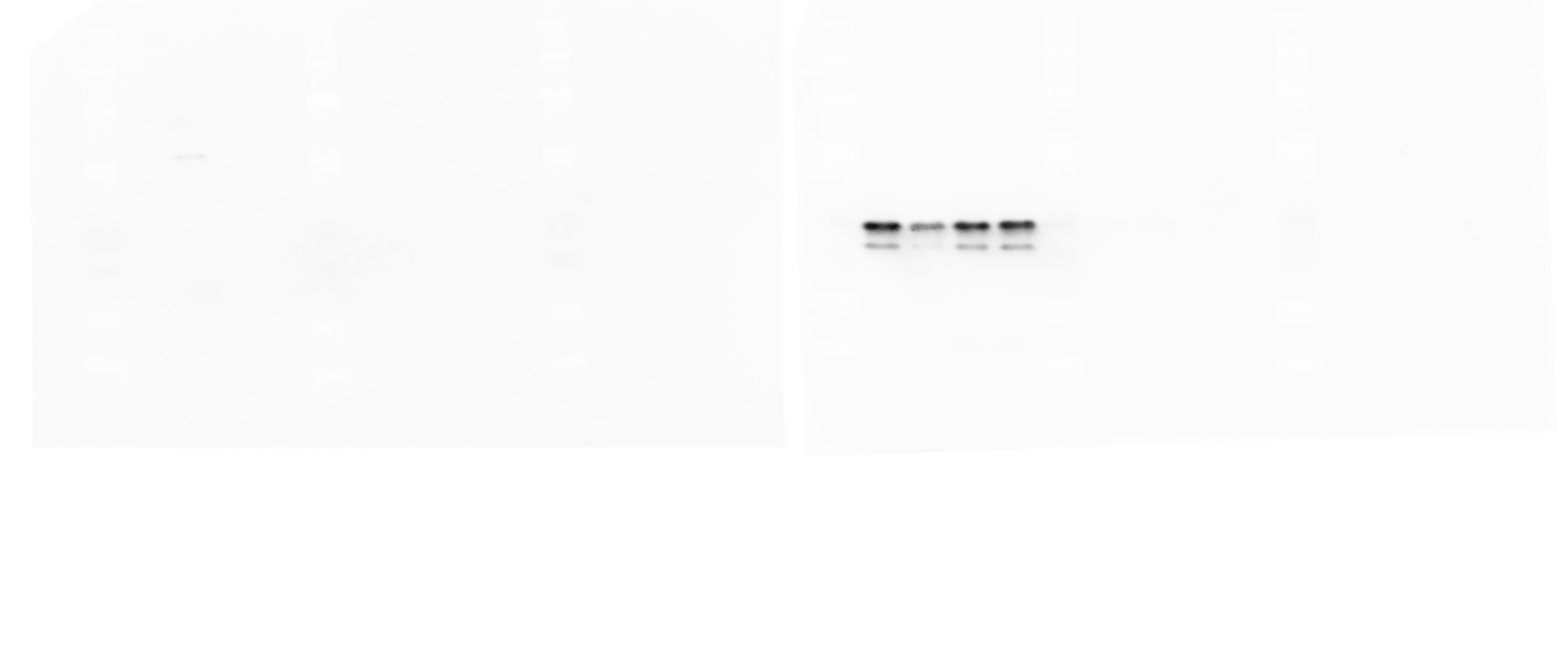

Supplement: Figure 4—source data 2. [file elife-98665-fig4-data2.zip › Figure 4-Source Data 2/Figure 4-Raw Gels-A.tif]

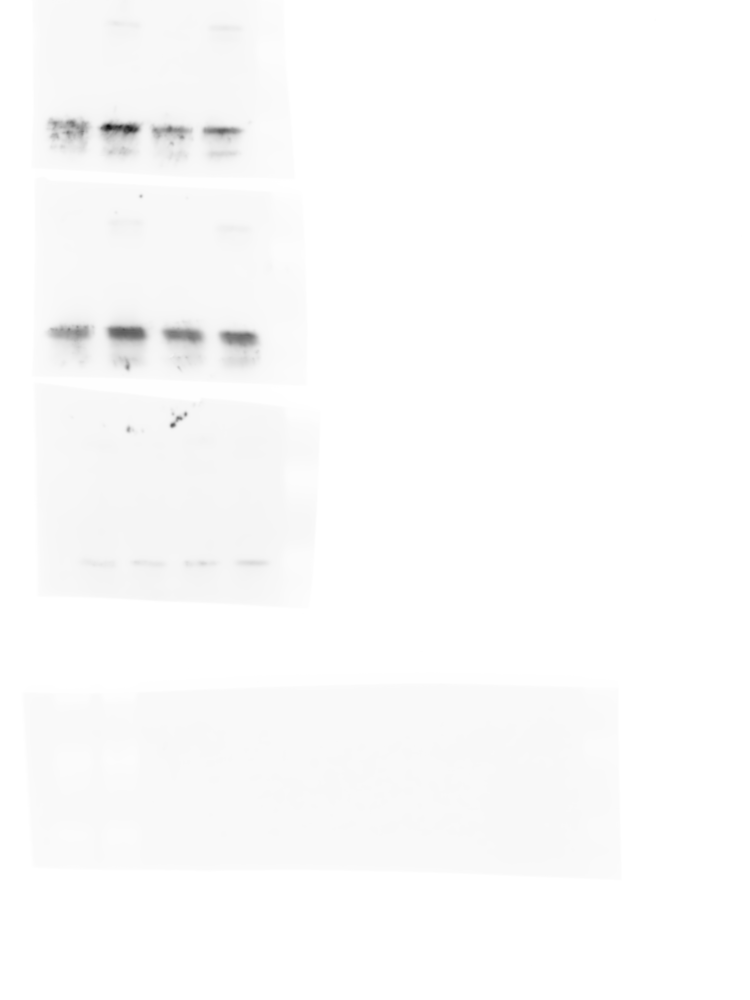

Supplement: Figure 4—figure supplement 1—source data 2. [file elife-98665-fig4-figsupp1-data2.zip › Figure 4-Figure Supplement 1-source data 2/Figure 4-Figure Supplement 1-Raw Gels-B.tif]

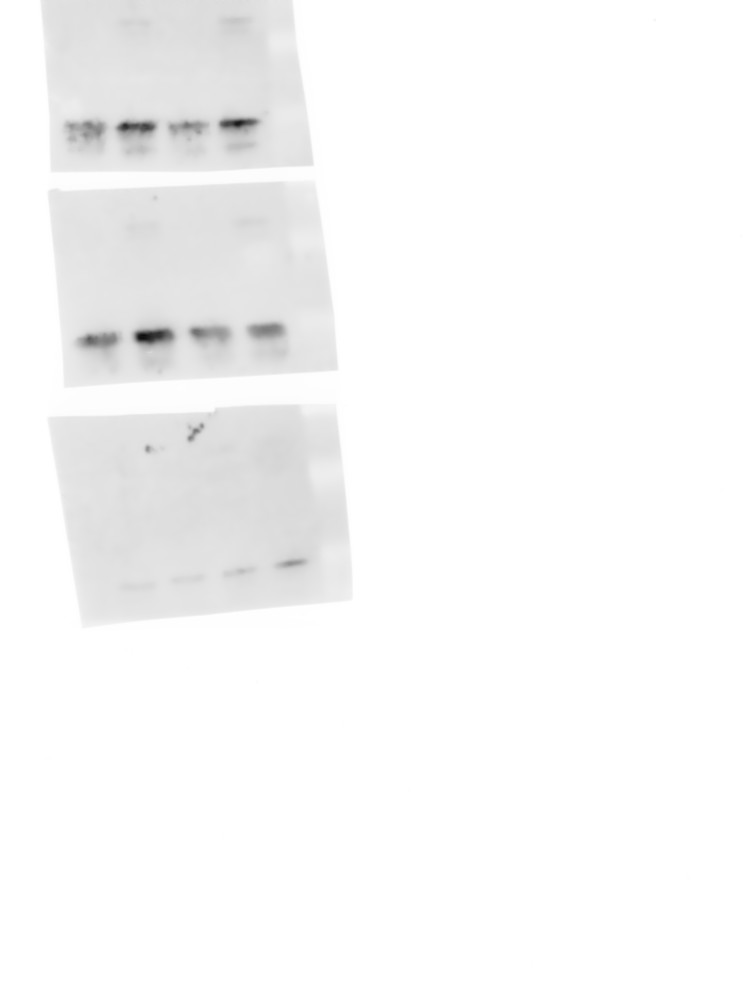

Supplement: Figure 4—figure supplement 1—source data 2. [file elife-98665-fig4-figsupp1-data2.zip › Figure 4-Figure Supplement 1-source data 2/Figure 4-Figure Supplement 1-Raw Gels-C.tif]

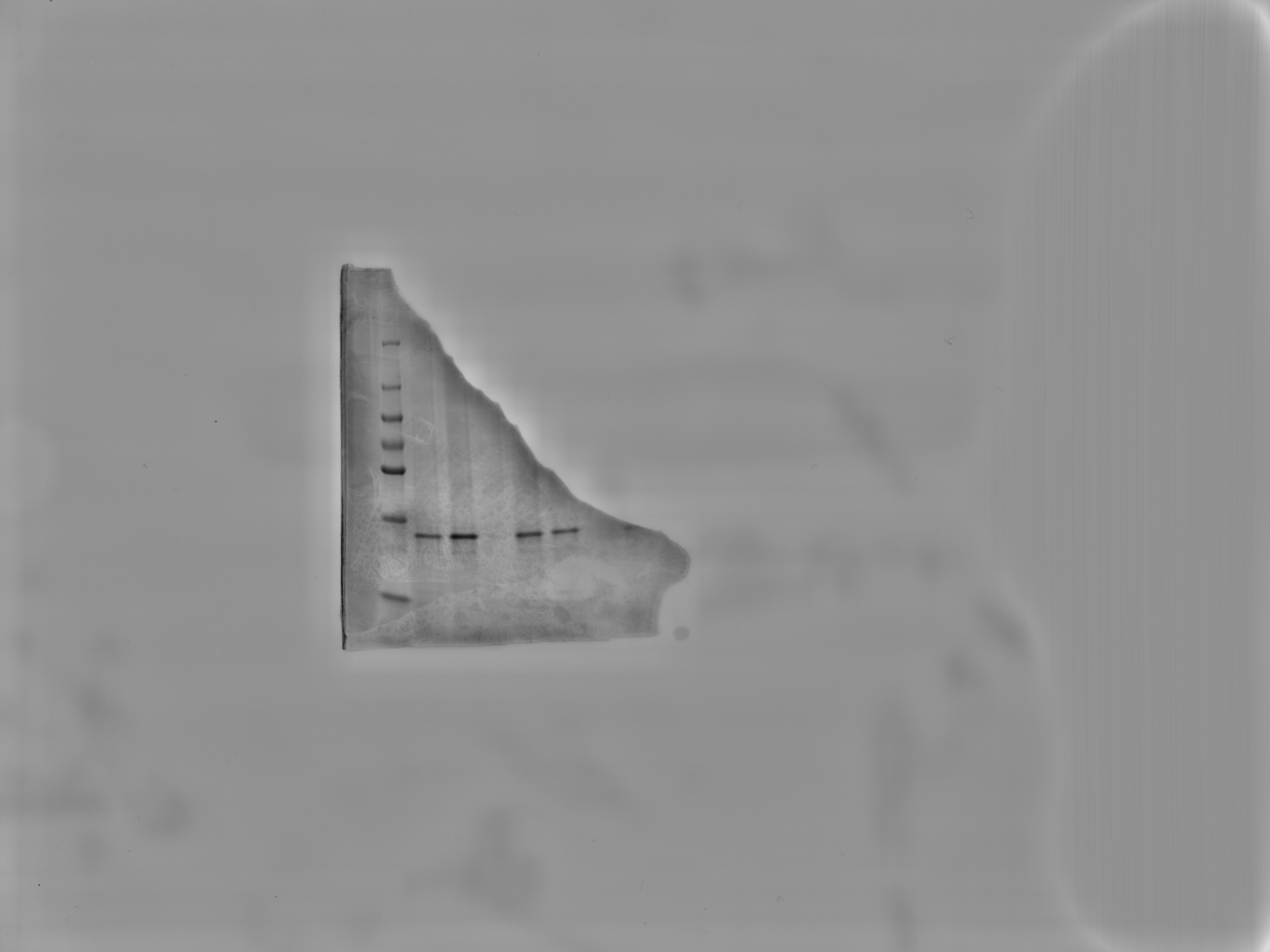

Supplement: Figure 4—figure supplement 1—source data 2. [file elife-98665-fig4-figsupp1-data2.zip › Figure 4-Figure Supplement 1-source data 2/Figure 4-Figure Supplement 1-Raw Gels-A.tif]
